# Supplementary material for: A multi-host mechanistic model of African swine fever emergence and control in Romania
Source: Nat Commun. 2026 Mar 30;17:2659. doi: 10.1038/s41467-026-70769-6 (PMC13035907; doi:10.1038/s41467-026-70769-6)
Supplement: Supplementary file 1 — Supplementary Information [file 41467_2026_70769_MOESM1_ESM.pdf]

Supplementary information for: A multi-host mechanistic model of African swine fever emergence and control in Romania

Brandon Hayes[1,2*], Timothée Vergne[1†], Nicolas Rose[2], Cristian Mortasivu[3], Mathieu Andraud[2†]

[1] Univ Toulouse, ENVT, INRAE, IHAP, Toulouse, France

[2] Ploufragan-Plouzané-Niort Laboratory, the French Agency for Food, Environmental and Occupational Health & Safety (ANSES), Ploufragan, France

[3] Romanian National Veterinary Sanitary and Food Safety Authority (ANSVSA), Bucharest, Romania

*Corresponding author

†These authors jointly supervised this work

Email: brandon.hayes@envt.fr

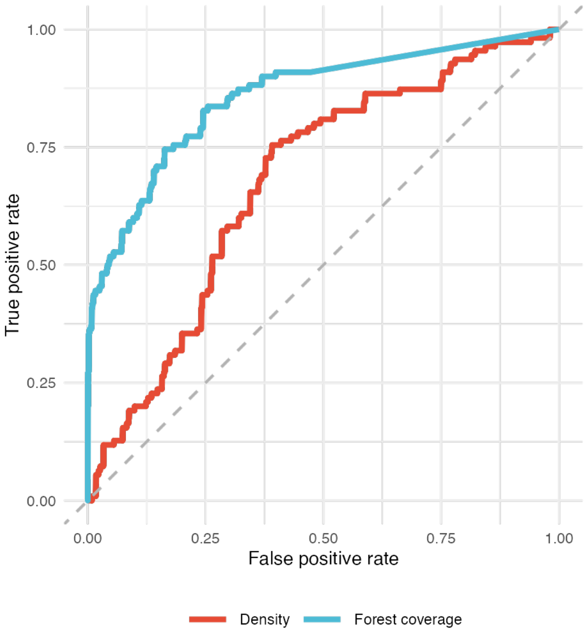

Supplementary Fig. 1. Selection of forest coverage as a habitat-based predictor for ASF risk. The receiver operating characteristic (ROC) curve of the performance of mean estimated wild boar density (red) and forest coverage proportion (blue) for predicting case occurrence at the level of the habitat patch is shown. Forest coverage, with an area under the curve (AUC) of 0.858, outperformed wild boar density (AUC = 0.677), supporting its suitability as a habitat-based predictor of ASF risk. Forest coverage was therefore selected as the preferred proxy, using an optimal threshold of 10.5% as computed via Youden's index.

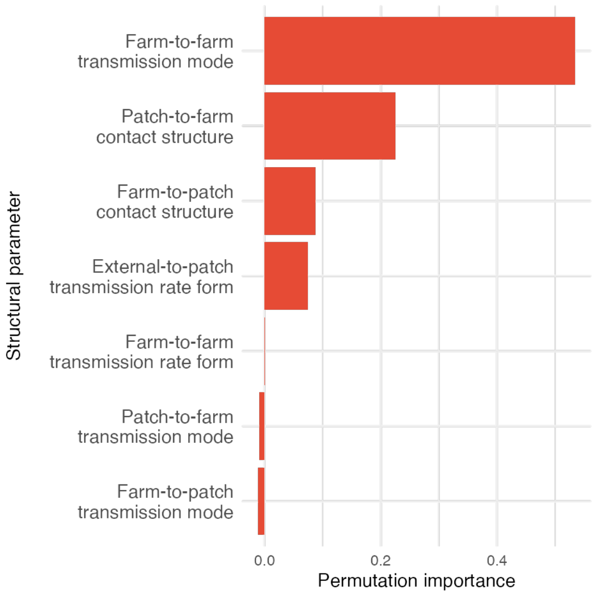

Supplementary Fig. 2. Permutation feature importance of structural model parameters. Farm-to-farm transmission mode and the contact structure between patches and farms had the greatest impact on final model fit. Conversely, the interspecific transmission mode between farms and patches, along with the form of the farm-to-farm transmission rate, had the least influence.

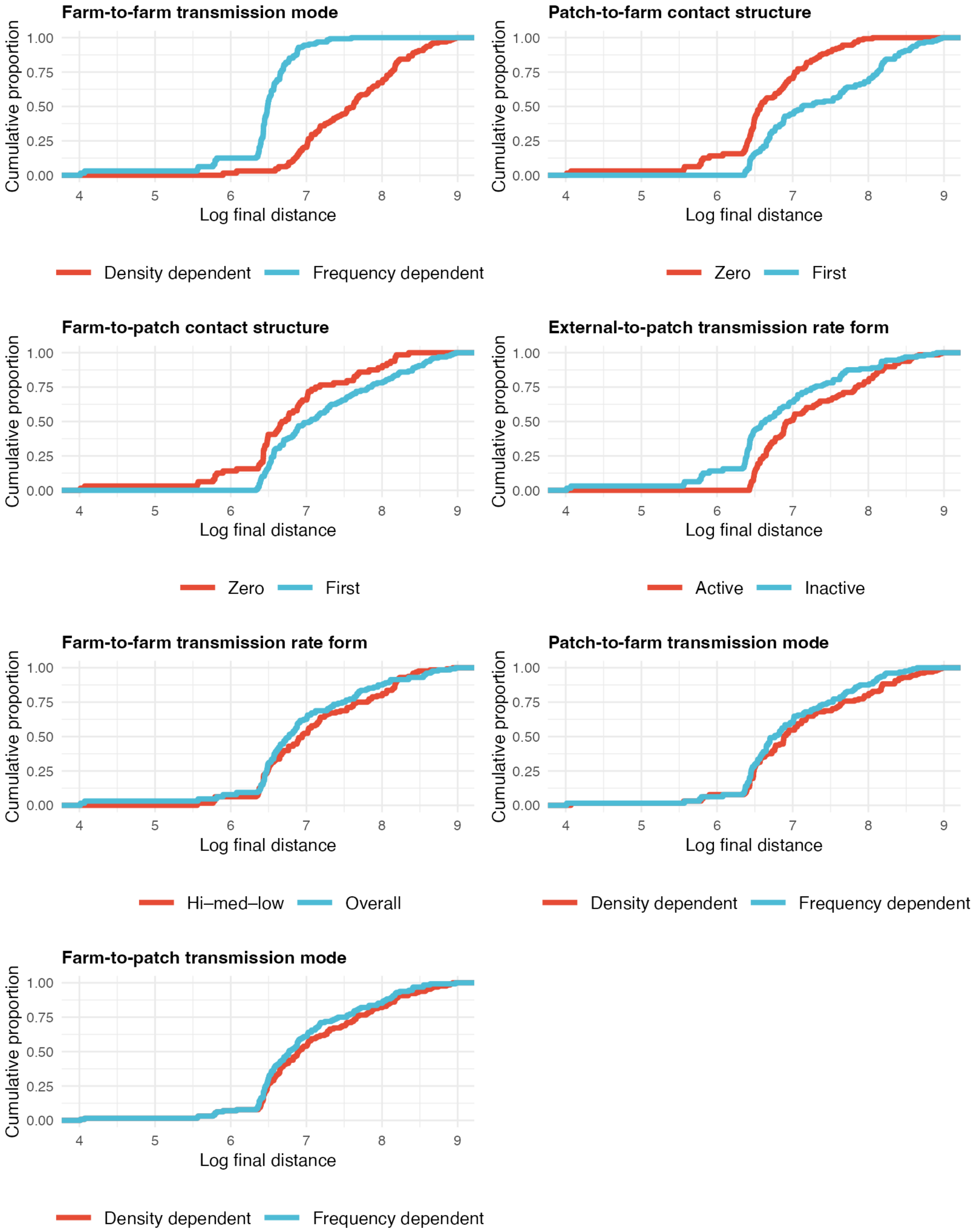

Supplementary Fig. 3. Comparative model fit across alternative structural assumptions. Here the empirical cumulative distribution functions for model fit characteristics per structural parameter formulation are provided. Each panel compares structural assumptions for a given structural parameter, using the log of the final distance between simulated and observed summary statistics (log final distance) to assess fit. For example, concerning the structural parameter for farm-to-farm transmission mode, models assuming frequency-dependent transmission (blue line) consistently outperformed those using density-dependent transmission (red line), with 89% of the models that used a frequency-dependent form having a log final distance below 7, in contrast to those that used density-dependent formulations.

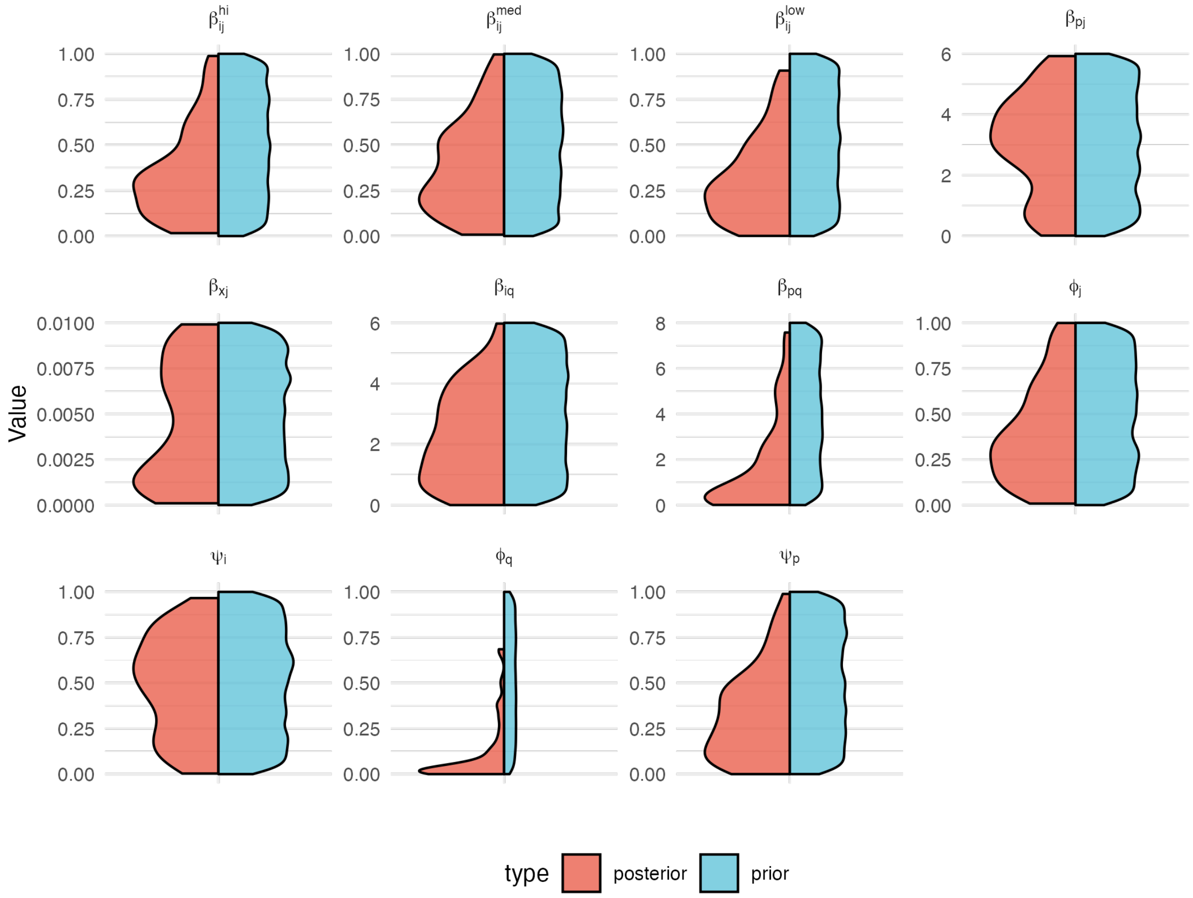

Supplementary Fig. 4. Prior and posterior distributions of conserved transmission parameters. Prior and posterior parameter distributions of conserved parameters were estimated from 2600 simulations through approximate Bayesian computation using summary statistics of weekly incidence by host and county. The final conserved parameter set consists of transmission rate terms for local spread: β_{ij}^{hi}, β_{ij}^{med}, and β_{ij}^{low} governing county-specific high-, medium-, and low-intensity transmission between farms, respectively; β_{pj}, representing transmission from domestic pig farms to wild boar patches; β_{xj} for external forces of infection acting on domestic pig farms; and β_{iq} and β_{pq}, governing transmission from wild boar patches to domestic pig farms and other wild boar patches, respectively. Parameters ϕ_j and ϕ_q represent relative susceptibility modifiers for domestic pig farms and wild boar patches, while ψ_i and ψ_p represent relative infectivity modifiers for domestic pig farms and wild boar patches.

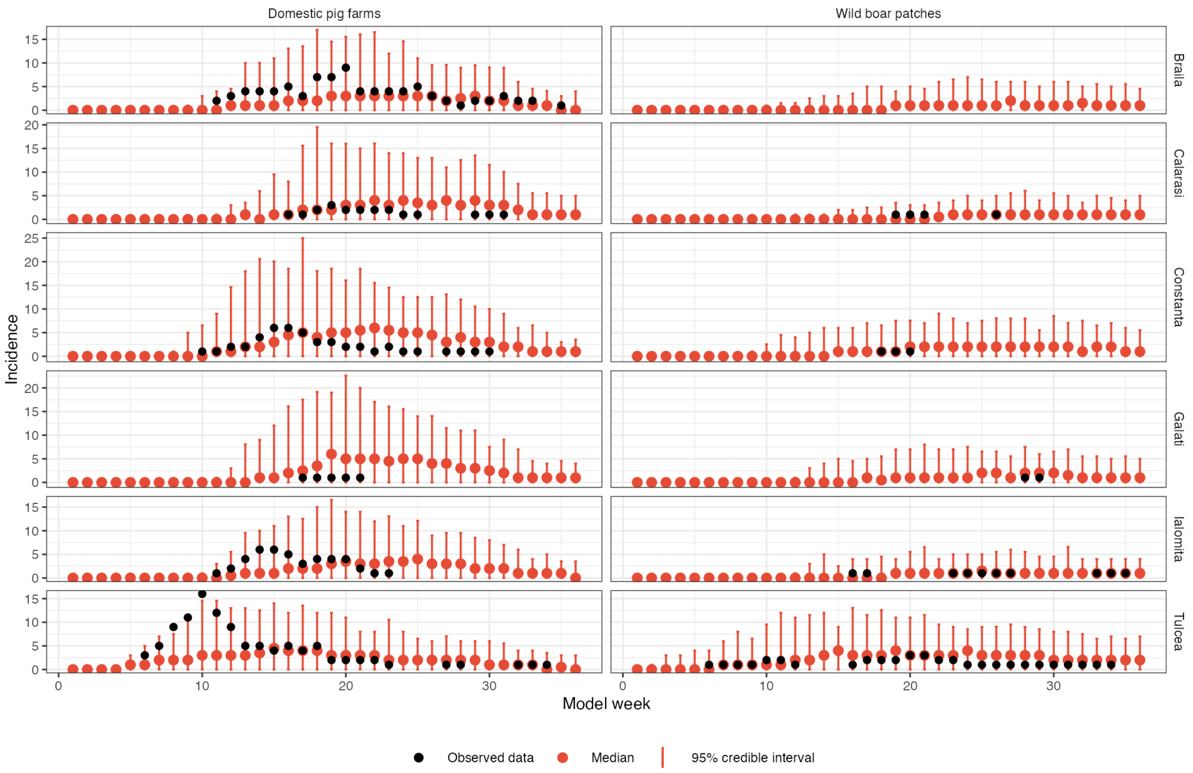

Supplementary Fig. 5. Model predictions against summary statistics. The outcomes of the best-fitting model across 500 simulations to observed weekly incidence in domestic pig farms and wild boar patches for each county, corresponding to 432 summary statistics (36 weeks by 6 counties by 2 host types). Reflecting weekly incidence by host and county, the 95% credible intervals (colored bar and point) are compared to the observed data (black point).

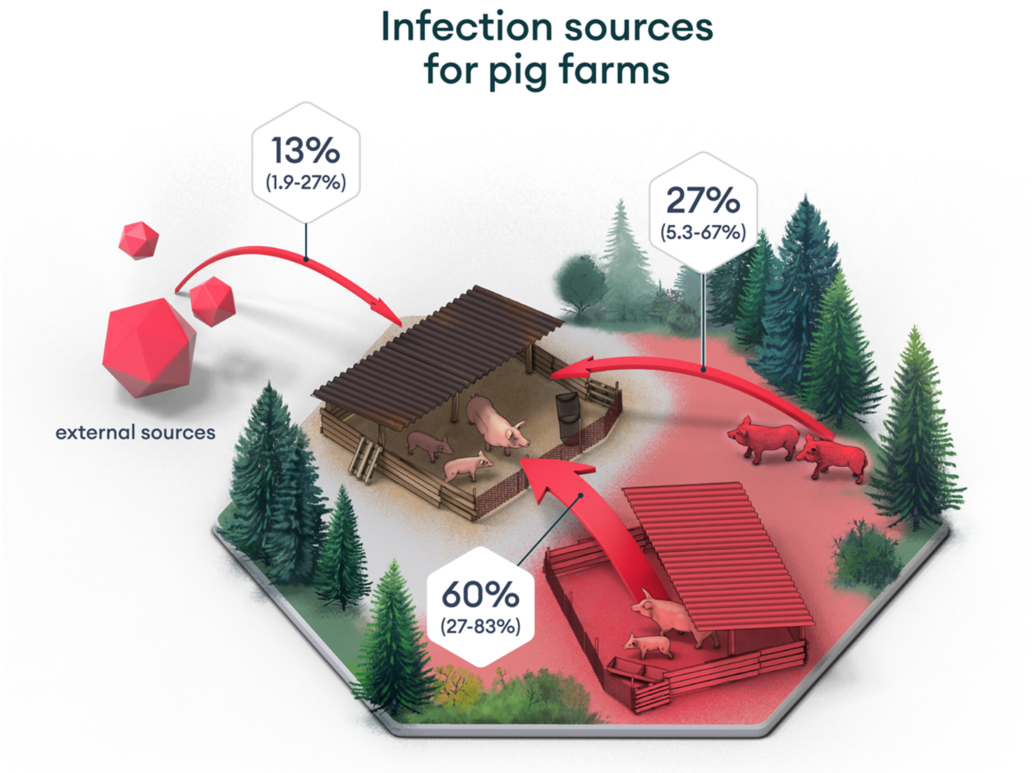

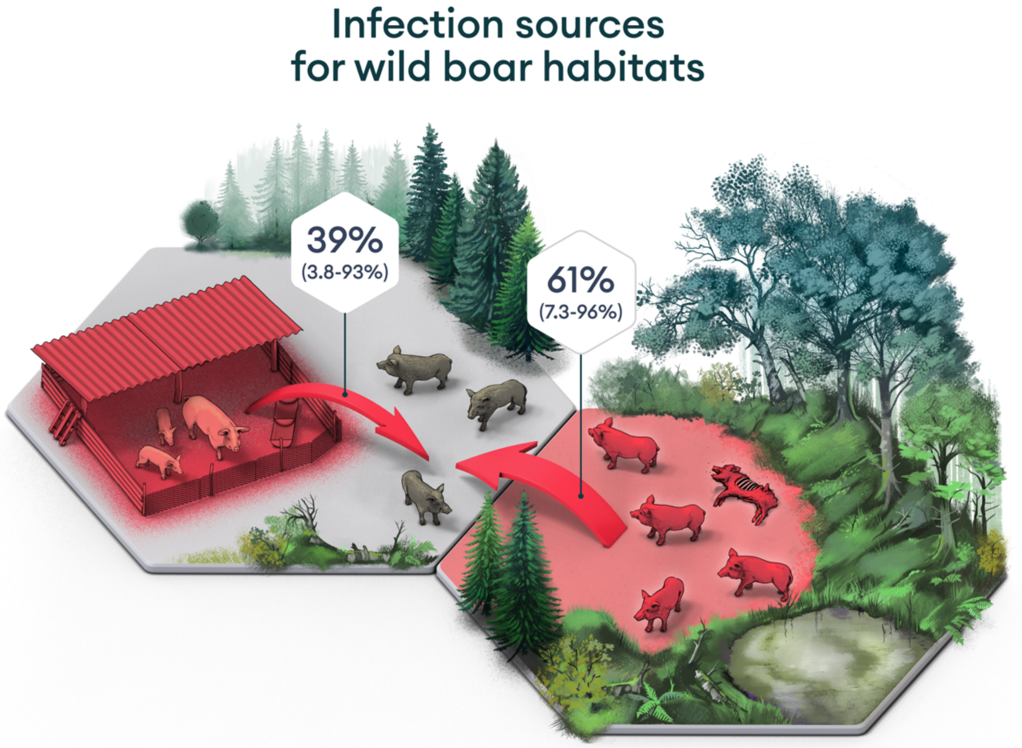

Supplementary Fig. 6. Graphical summary of infection source estimation. Proportional contribution of each transmission pathway, summarized through posterior means and 95% credible intervals, to infections among domestic pig farms (top) and wild boar habitat patches (bottom) as estimated from the best-fitting model. Arrows indicate the direction of transmission, with red shading indicating source infectiousness. Graphic design by Pierre Bourcier.

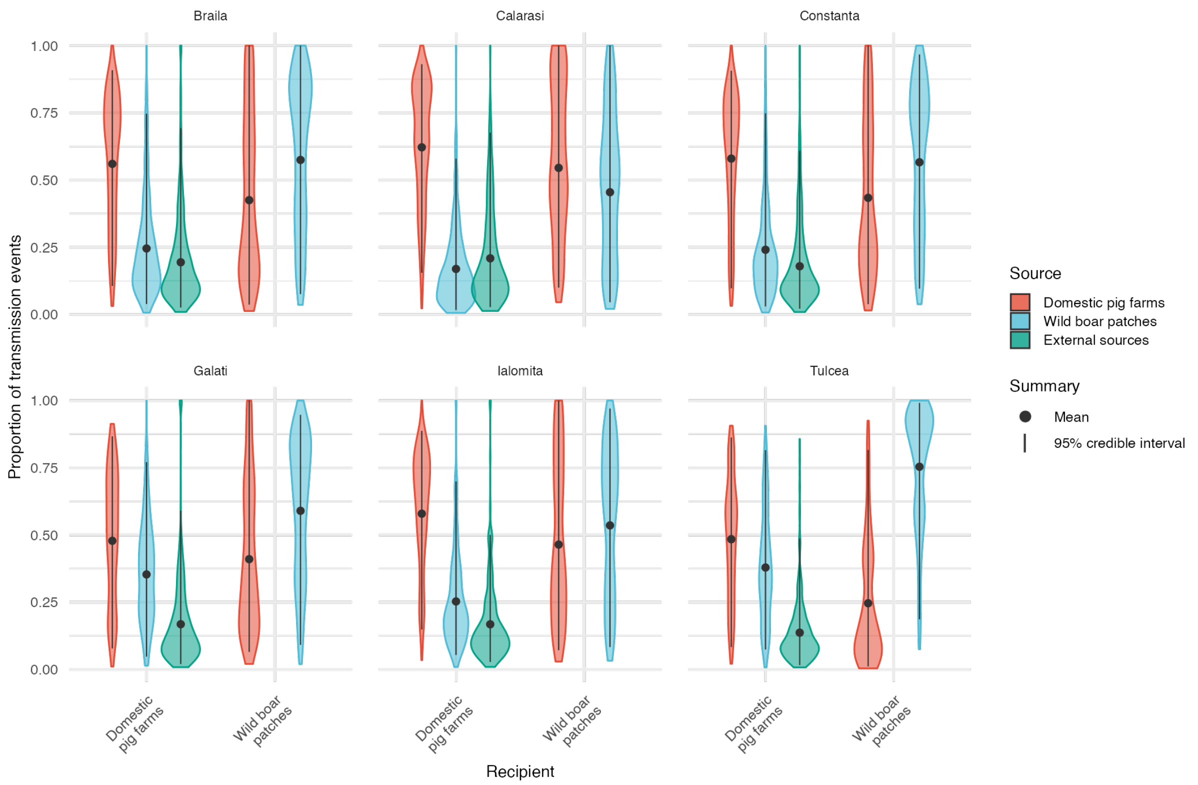

Supplementary Fig. 7. County-level contributions of transmission pathways. Transmission pathway contribution to overall epidemic dynamics, based on 500 simulations of the best-fitting model, was faceted by county. Similar to the overall results (Figure 2), the majority of domestic pig cases originated from other farms, while wild boar infections were primarily driven by transmission among wild boar. Notably Tulcea county, the most rural county and home to the Danube Delta, experienced the highest estimated contribution from wild boar sources, with a mean of 75% (19–99%) of wild boar and 38% (7.4–82%) of domestic pig cases attributed to wild boar.

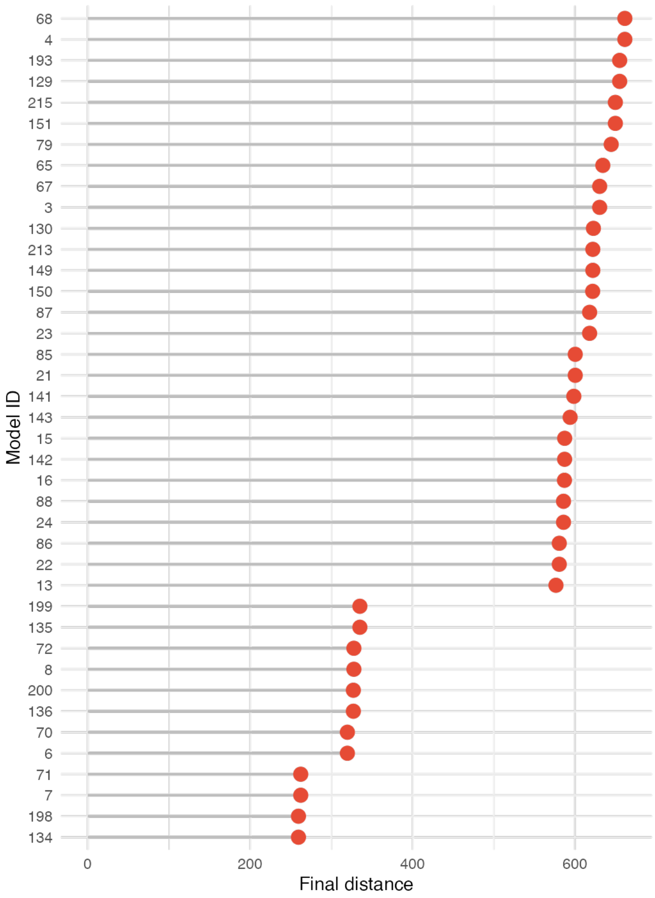

Supplementary Fig. 8. Model ranking by final distance and identification of top-performing variants. The 40 models that fulfilled the inclusion criteria of capturing the observed epidemic trajectory in at least four counties were ranked by their final total Euclidian distance from the summary statistics. Lower values indicate a better fit. Three performance groups are noted, with a subset of 12 models forming a cluster at the lowest distances. This subset was subsequently selected for the ensemble analysis.

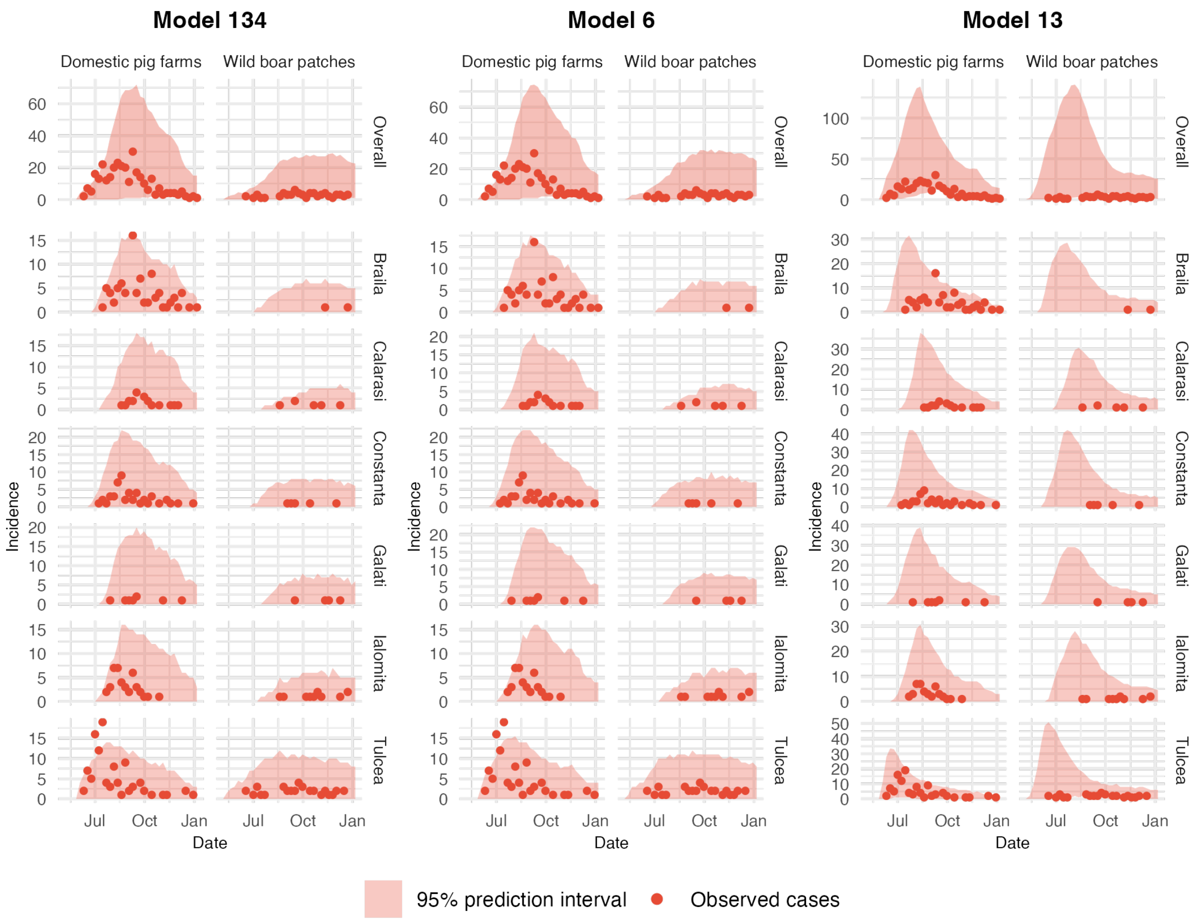

Supplementary Fig. 9. Representative incidence trajectories across model performance clusters. Observed and predicted weekly incidence of the lowest-distance model in each cluster identified in Supplementary Fig. 7, representative of the trajectories of other models within the same cluster. Shaded ribbons represent 95% prediction intervals of the best-fitting model (determined across 500 simulations of 100 conserved particles), and points indicate observed case counts. The first two numerical clusters, comprising 12 models and represented by model 134 and model 6, respectively, exhibit similar trajectories with the second group having a slightly poorer fit in Galati and Tulcea. Beyond these first 12 models, results start to diverge from observed trajectories, notably among estimated wild boar incidence, as seen with Model 13. Consequently, the first two numerical clusters were treated as a single group for ensemble analysis.

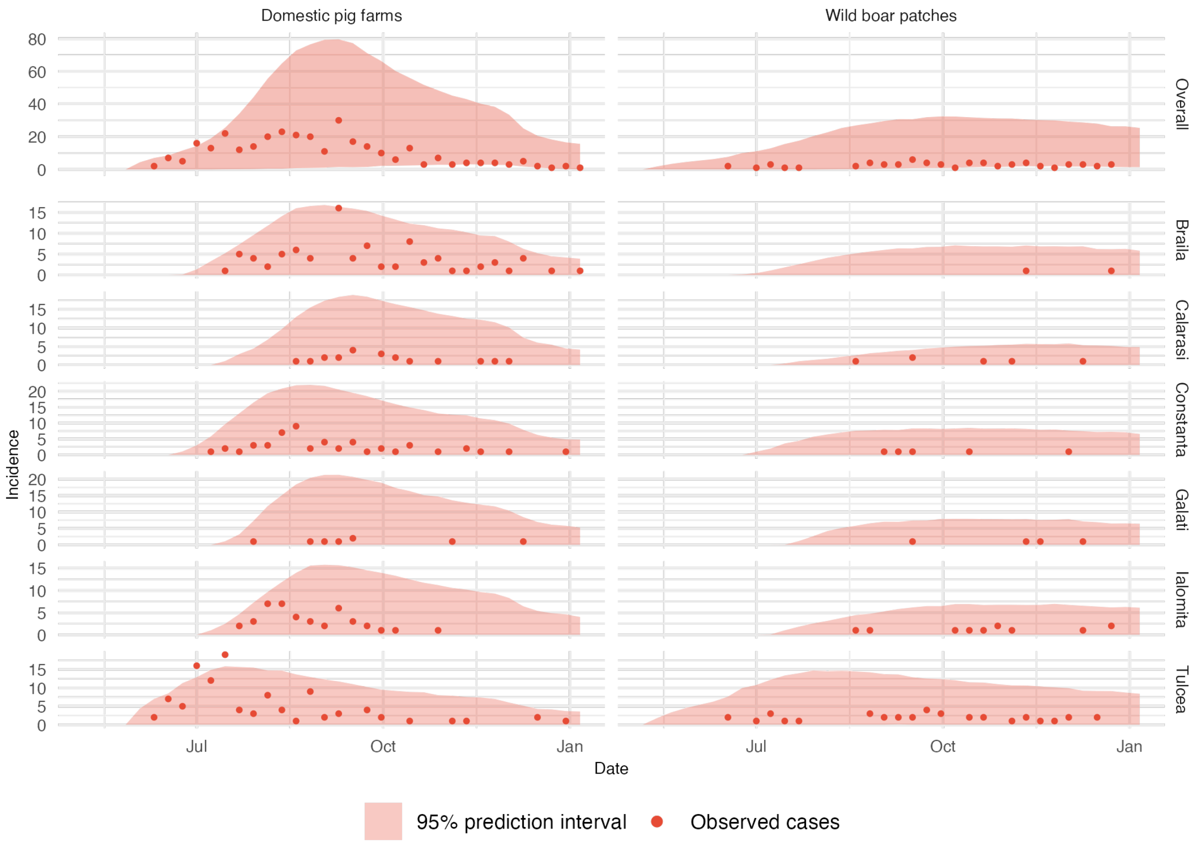

Supplementary Fig. 10. Ensemble-based weekly incidence predictions by host and county. Predicted weekly incidence trajectories in domestic pig farms and wild boar patches obtained from a performance-weighted ensemble of the 12 best-fitting model variants. Ensemble trajectories were constructed via model averaging, with individual model contributions weighted by their relative goodness-of-fit to the observed summary statistics. Shaded ribbons represent 95% prediction intervals of the ensemble estimates and points indicate observed case counts. The ensemble model maintained the predicted timing and magnitude of the best-fit single-model results (presented in Figure 1), though with wider uncertainty bands.

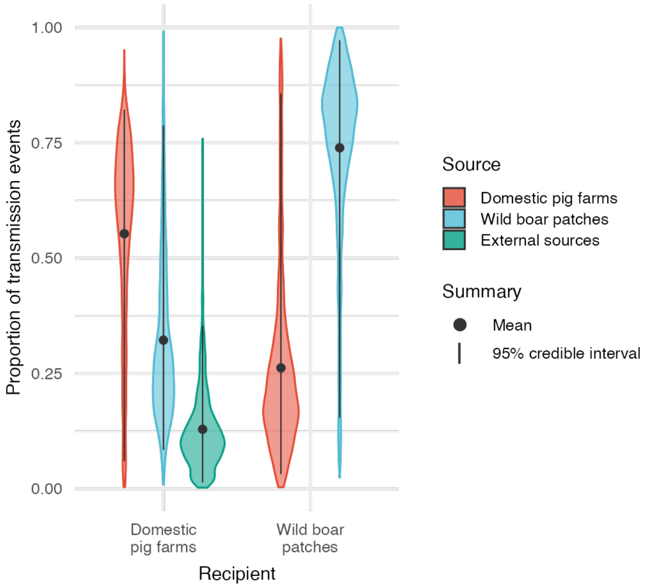

Supplementary Fig. 11. Transmission pathway contributions from the weighted ensemble. Transmission pathway contributions to overall epidemic propagation were computed from the weighted ensemble of the top 12 model variants. For each recipient (x-axis), the distribution of the proportion of infections attributed to each source are shown. The central dot and bar indicate the mean and 95% credible intervals (CI_{95}). Domestic pig farms were most frequently infected by other domestic pig farms (mean: 55%, CI_{95}: 6.0–82%), followed by wild boar patches (32%, 8.4–79%) and external sources (13%, 1.4-35%). Conversely, most infections in wild boar patches originated from other wild boar patches (74%, 15–97%), and a smaller proportion came from domestic pig farms (26%, 3.1–86%).

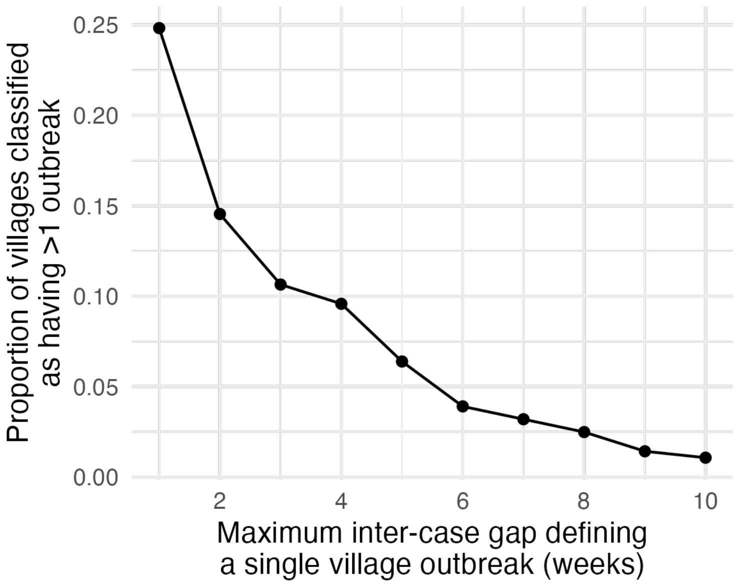

Supplementary Fig. 12. Sensitivity of village-level outbreak classification to outbreak window definition. The proportion of villages classified as having more than one outbreak is shown as a function of the maximum time gap that was allowed between consecutive detected cases within a village to define a single outbreak. An elbow heuristic identified 3 weeks as the most parsimonious value prior to diminishing returns.

Supplementary Table 1. Sensitivity of final epidemic size to increased detection rates within surveillance zones (SZ), in comparison to the baseline scenario which had assumed a 1.5x multiplier.

Scenario	Host	Median difference (vs. baseline)	95% Credible interval	P(< baseline)
1x multiplier within SZ	Dom. pig farms	-3	-658–670	0.503
	Wild boar patches	0	-332–341	0.498
2x multiplier within SZ	Dom. pig farms	1	-656–653	0.498
	Wild boar patches	1	-335–339	0.496
3x multiplier within SZ	Dom. pig farms	-5	-659–646	0.506
	Wild boar patches	-4	-345–330	0.507

Supplementary Table 2. Alternative formulations of key structural parameters

Component	Transmission pathway	Formulation	Equation modification
Contact structure	Farm-to-patch	Zero-order / first-order	Eq. 2: $\mathbb{1}_{ab_{contact}}$ expanded to include patches neighboring the patch that contains the farm
	Patch-to-farm	Zero-order / first-order	Eq. 2: $\mathbb{1}_{ab_{contact}}$ expanded to include farms in neighboring patches
Transmission mode	Farm-to-farm	Density / frequency dependent	Eq. 2: $\lambda_{ij} \rightarrow \lambda_{ij}/N$ N = number of farms in contact
	Patch-to-farm	Density / frequency dependent	Eq. 2: $\lambda_{pj} \rightarrow \lambda_{pj}/N$ N = number of farms in contact
	Farm-to-patch	Density / frequency dependent	Eq. 2: $\lambda_{iq} \rightarrow \lambda_{iq}/N$ N = number of patches in contact
Transmission rate	Farm-to-farm	Overall / high-medium-low	Eq. 2: β_{ij} varies by county risk profile
	Patch-to-patch	Overall / high-low	Eq. 2: β_{pq} varies by county risk profile
	External-to-patch	Active / inactive	Eq. 3: $\beta_{xq} > 0 \rightarrow \beta_{xq} = 0$
