# Supplementary material for: A multi-host mechanistic model of African swine fever emergence and control in Romania
Source: Nat Commun. 2026 Mar 30;17:2659. doi: 10.1038/s41467-026-70769-6 (PMC13035907; doi:10.1038/s41467-026-70769-6)
Supplement: Supplementary file 2 — Reporting Summary [file 41467_2026_70769_MOESM2_ESM.pdf]

nature portfolio

Corresponding author(s): Brandon Hayes

Last updated by author(s): Dec 10, 2025

Reporting Summary

Nature Portfolio wishes to improve the reproducibility of the work that we publish. This form provides structure for consistency and transparency in reporting. For further information on Nature Portfolio policies, see our Editorial Policies and the Editorial Policy Checklist.

Statistics

For all statistical analyses, confirm that the following items are present in the figure legend, table legend, main text, or Methods section.

n/a	Confirmed	
☐	☒	The exact sample size (*n*) for each experimental group/condition, given as a discrete number and unit of measurement
☐	☒	A statement on whether measurements were taken from distinct samples or whether the same sample was measured repeatedly
☒	☐	The statistical test(s) used AND whether they are one- or two-sided<br>*Only common tests should be described solely by name; describe more complex techniques in the Methods section.*
☐	☒	A description of all covariates tested
☐	☒	A description of any assumptions or corrections, such as tests of normality and adjustment for multiple comparisons
☐	☒	A full description of the statistical parameters including central tendency (e.g. means) or other basic estimates (e.g. regression coefficient) AND variation (e.g. standard deviation) or associated estimates of uncertainty (e.g. confidence intervals)
☒	☐	For null hypothesis testing, the test statistic (e.g. *F*, *t*, *r*) with confidence intervals, effect sizes, degrees of freedom and *P* value noted<br>*Give P values as exact values whenever suitable.*
☐	☒	For Bayesian analysis, information on the choice of priors and Markov chain Monte Carlo settings
☒	☐	For hierarchical and complex designs, identification of the appropriate level for tests and full reporting of outcomes
☒	☐	Estimates of effect sizes (e.g. Cohen's *d*, Pearson's *r*), indicating how they were calculated

Our web collection on statistics for biologists contains articles on many of the points above.

Software and code

Policy information about availability of computer code

Data collection	No software was used to collect data
Data analysis	Open-source R software (v. 4.3.3) was used for all analysis, and code is available through the public-facing GitLab repository: https://gitlab.envt.fr/epidesa/asf-multihost-romania. Model calibration was performed via the EasyABC package, random forest feature selection was performed via the ranger package, and data manipulation and visualization was performed via the tidyverse suite.

For manuscripts utilizing custom algorithms or software that are central to the research but not yet described in published literature, software must be made available to editors and reviewers. We strongly encourage code deposition in a community repository (e.g. GitHub). See the Nature Portfolio guidelines for submitting code & software for further information.

Data

Policy information about availability of data

All manuscripts must include a data availability statement. This statement should provide the following information, where applicable:

- Accession codes, unique identifiers, or web links for publicly available datasets
- A description of any restrictions on data availability
- For clinical datasets or third party data, please ensure that the statement adheres to our policy

Data can be accessed at: https://gitlab.envt.fr/epidesa/asf-multihost-romania

Research involving human participants, their data, or biological material

Policy information about studies with human participants or human data. See also policy information about sex, gender (identity/presentation), and sexual orientation and race, ethnicity and racism.

Reporting on sex and gender	No human participants were involved in this research
Reporting on race, ethnicity, or other socially relevant groupings	*Please specify the socially constructed or socially relevant categorization variable(s) used in your manuscript and explain why they were used. Please note that such variables should not be used as proxies for other socially constructed/relevant variables (for example, race or ethnicity should not be used as a proxy for socioeconomic status).*<br>*Provide clear definitions of the relevant terms used, how they were provided (by the participants/respondents, the researchers, or third parties), and the method(s) used to classify people into the different categories (e.g. self-report, census or administrative data, social media data, etc.)*<br>*Please provide details about how you controlled for confounding variables in your analyses.*
Population characteristics	*Describe the covariate-relevant population characteristics of the human research participants (e.g. age, genotypic information, past and current diagnosis and treatment categories). If you filled out the behavioural & social sciences study design questions and have nothing to add here, write "See above."*
Recruitment	*Describe how participants were recruited. Outline any potential self-selection bias or other biases that may be present and how these are likely to impact results.*
Ethics oversight	*Identify the organization(s) that approved the study protocol.*

Note that full information on the approval of the study protocol must also be provided in the manuscript.

Field-specific reporting

Please select the one below that is the best fit for your research. If you are not sure, read the appropriate sections before making your selection.

☐ Life sciences ☐ Behavioural & social sciences ☒ Ecological, evolutionary & environmental sciences

For a reference copy of the document with all sections, see nature.com/documents/nr-reporting-summary-flat.pdf

Ecological, evolutionary & environmental sciences study design

All studies must disclose on these points even when the disclosure is negative.

Study description	This study is a retrospective analysis of existing surveillance datasets that describe African swine fever (ASF) detections in domestic pigs (Sus scrofa domesticus) and wild boar (Sus scrofa) in Romania. Investigation occurred through a spatially explicit, stochastic, multi-host transmission model parameterized to the first wave of the current epidemic (observed June–December 2018).<br><br>This quantitative study is simulation-based, where epidemiological assumptions and control strategies are treated as treatment factors. A factorial simulation design was used to examine the interactions between and combined effects of treatment factors across 256 distinct model combinations. The experimental unit is a single stochastic simulation realization. Model calibration required 2600 replications to achieve a set of 100 conserved particles under a sequential Monte Carlo process. For evaluating control strategies, 500 stochastic simulation replicates were run per strategy to characterize outcome variability across epidemic scenarios.
Research sample	The research sample consists of national surveillance records of ASF detections in domestic pigs (Sus scrofa domesticus) and wild boar (Sus scrofa) in Romania. These records include all reported ASF outbreaks during the first epidemic wave (June–December 2018). Individual-level attributes, such as sex or age, were not available in the surveillance data nor were they required for the territorial-scale epidemic modelling that was performed.<br><br>No live organisms were manipulated for this study.<br><br>This sample is intended to represent the national domestic pig and wild boar populations that contributed to the spread of ASF during the early phase of the Romanian epidemic. This data served as the empirical basis for designing, parameterizing, and validating the spatially-explicit, multi-host simulation model.
Sampling strategy	No sampling was performed, rather all available national surveillance data that met the inclusion criteria were used. Consequently, no a priori sample size calculation was deemed necessary. In our study, sample size was determined by the total number of reported ASF cases, and was sufficient to capture the observed spatiotemporal epidemic patterns that were required for model parameterization.
Data collection	ASF case data were originally recorded by Romanian national veterinary authorities following standard operating procedures. This data was then aggregated and published by the World Organization for Animal Health (WOAH). The study authors then retrieved that data using the WOAH Animal Disease Information System (formerly Animal Disease Notification System).
Timing and spatial scale	The dataset covers ASF detections occurring between June 1, 2018 and December 31, 2018 for the 6 counties in southeastern

Timing and spatial scale	Romania, corresponding to the first epidemic wave in Romania. The data reporting frequency is event-based, with each detection associated with a specific date and GPS-tagged location. No cohort structure was defined beyond routine surveillance.
Data exclusions	No data were excluded; all reported ASF detections in the study period within the region of study were used in the study
Reproducibility	All modelling scripts and data are openly available, and testing of the full model pipeline on a clean system reproduced the reported results with expected minor variation, due to the stochastic nature of the model.
Randomization	Not applicable: this study employed stochastic simulations to reproduce epidemic trajectories through the use of retrospective surveillance data.
Blinding	Not applicable: no personally identifiable information was used nor could previous knowledge of the used datasets influence the outcomes of the stochastic epidemic simulations.

Did the study involve field work? ☐ Yes ☒ No

Reporting for specific materials, systems and methods

We require information from authors about some types of materials, experimental systems and methods used in many studies. Here, indicate whether each material, system or method listed is relevant to your study. If you are not sure if a list item applies to your research, read the appropriate section before selecting a response.

Materials & experimental systems

n/a	Involved in the study	
☒	☐	Antibodies
☒	☐	Eukaryotic cell lines
☒	☐	Palaeontology and archaeology
☒	☐	Animals and other organisms
☒	☐	Clinical data
☒	☐	Dual use research of concern
☒	☐	Plants

Methods

n/a	Involved in the study	
☒	☐	ChIP-seq
☒	☐	Flow cytometry
☒	☐	MRI-based neuroimaging

Plants

Seed stocks	*Report on the source of all seed stocks or other plant material used. If applicable, state the seed stock centre and catalogue number. If plant specimens were collected from the field, describe the collection location, date and sampling procedures.*
Novel plant genotypes	*Describe the methods by which all novel plant genotypes were produced. This includes those generated by transgenic approaches, gene editing, chemical/radiation-based mutagenesis and hybridization. For transgenic lines, describe the transformation method, the number of independent lines analyzed and the generation upon which experiments were performed. For gene-edited lines, describe the editor used, the endogenous sequence targeted for editing, the targeting guide RNA sequence (if applicable) and how the editor was applied.*
Authentication	*Describe any authentication procedures for each seed stock used or novel genotype generated. Describe any experiments used to assess the effect of a mutation and, where applicable, how potential secondary effects (e.g. second site T-DNA insertions, mosiacism, off-target gene editing) were examined.*
